# Protective effects of *Bacillus licheniformis* on growth performance, gut barrier functions, immunity and serum metabolome in lipopolysaccharide-challenged weaned piglets

**DOI:** 10.3389/fimmu.2023.1140564

**Published:** 2023-03-22

**Authors:** Xiaorong Yu, Zhenglie Dai, Guangtian Cao, Zhenchuan Cui, Ruiqiang Zhang, Yinglei Xu, Yanping Wu, Caimei Yang

**Affiliations:** ^1^ College of Animal Science and Technology, College of Veterinary Medicine, Zhejiang Agricultural and Forestry University, Hangzhou, China; ^2^ College of Standardisation, China Jiliang University, Hangzhou, China

**Keywords:** *Bacillus licheniformis*, lipopolysaccharides, growth performance, gut barrier functions, immunity, serum metabolome

## Abstract

*Bacillus licheniformis* (*B. licheniformis*) is a well-accepted probiotic that has many benefits on both humans and animals. This study explored the effects of *B. licheniformis* on growth performance, intestinal mucosal barrier functions, immunity as well as serum metabolome in the weaned piglets exposed to lipopolysaccharide (LPS). One hundred and twenty piglets weaned at four weeks of age were separated into two groups that received a basal diet (the control group, CON), and a basal diet complemented with *B. licheniformis* (500 mg/kg, the BL group, BL). Twenty-four piglets were chosen from the above two groups and 12 piglets were injected with LPS intraperitoneally at a concentration of 100 μg/kg and the others were injected with sterile saline solution of the same volume. All the piglets were sacrificed 4 h after LPS challenge. Results showed that *B. licheniformis* enhanced the ADG and final body weight and lowered the F/G and diarrhea rate. Pre-treatment with *B. licheniformis* markedly attenuated intestinal mucosal damage induced by LPS challenge. Supplementation with *B. licheniformis* strengthened immune function and suppressed inflammatory response by elevating the concentrations of serum immunoglobulin (Ig) A and jejunum mucosal IgA and IgG and decreasing serum IL-6 and jejunum mucosal IL-1β. In addition, *B. licheniformis* pretreatment prevented LPS-induced intestinal injury by regulating the NLRP3 inflammasome. Furthermore, pretreatment with *B. licheniformis* tended to reverse the reduction of acetate and propionic acids in the colonic contents that occurred due to LPS stress. *B. licheniformis* markedly modulated the metabolites of saccharopine and allantoin from lysine and purine metabolic pathways, respectively. Overall, these data emphasize the potentiality of *B. licheniformis* as a dietary supplement to overcome the challenge of bacterial LPS in the animal and to enhance the food safety.

## 1 Introduction

Intestinal tract can not only digest and absorb nutrients, but also provide a protective barrier to maintain homeostasis (1). At the same time, intestinal tract is home to many microbes, which have many effects for nutrition, metabolism, and immunity (2). Healthy intestinal microorganisms and their metabolites play an important role in inhibiting pathogen infection, synthesizing volatile fatty acids (VFAs), regulating fat metabolism, and promoting immune system development (3, 4). It is worth noting that intestinal microorganisms can ferment host indigestible carbohydrates into VFAs, which may have anti-inflammatory and immunomodulatory effects (5). Intestinal microorganisms dysbiosis alters host physiological function, leading to chronic inflammatory and metabolic diseases (6). Thus, it is important to maintain health gut microbes to host health.

It is widely believed that probiotics are beneficial to intestinal health when administered in adequate amounts (7). Probiotics can perform various functions by colonizing the intestinal mucosal layer (8). Among the probiotic strains studied, two popular lipopeptide biosurfactants, plipastatin and surfactin of *B. licheniformis* have strong bactericidal capacities (9). Prior studies suggested that *B. licheniformis* positively affects the growth performance of pigs (10, 11). Furthermore, *B. licheniformis*-fermented products reduce the prevalence of diarrhea and change the fecal microbiota in the weaned piglets (12). A recent study reported that *B. licheniformis* supplementation could lead to better growth behavior, reduce diarrhea incidence, and increase the feed conversion ratio in piglets (13). Moreover, the ingestion of a blend of *Bacillus* probiotics could regulate the intestinal inflammation and keep the integrity of the intestinal epithelial barrier (14). Dietary compound probiotics containing *B. licheniformis* have beneficial effects on the growth performance, the digestibility of nutrients, and the fecal microbiota of the weaned piglets (15).

Lipopolysaccharide (LPS) is a primary outer membrane element of gram-negative bacteria that can cause inflammation, immune stress, and tissue damage in piglets (16, 17). The LPS stress is an excellent animal model for studying oxidative stress and intestinal injury (18). CSP32, a polypeptide isolated from a *Bacillus* strain, significantly diminished the generation of pro-inflammatory mediators in RAW 264.7 macrophages induced by LPS (19). Deng et al. (20) found that both *B. licheniformis* and *Bacillus subtilis* (*B. subtilis*) could promote intestinal growth, improve digestive enzyme activities, and alleviate LPS-induced injury in rats. Idowu et al. (21) reported a protective effect of supplemental feed additives containing *B. licheniformis* against LPS-induced inflammatory stress or tissue damage in new-weaned beef cattle. However, to our knowledge, there is no comprehensive study of *B. licheniformis* on growth performance, metabolomics, and intestinal health in pigs in an LPS-challenged model. We assumed that *B. licheniformis* might play a protective role against LPS stress. In the present study, we assessed the role of *B. licheniformis* in protecting the growth performance, incidence of diarrhea, immune function, intestinal morphology, serum metabolome and colonic VFAs in LPS-challenged weaned piglets.

## 2 Materials and methods

### 2.1 Animals and feedings

One hundred and twenty Duroc × Landry × Yorkshire piglets weaned at four weeks of age were assigned to two dietary treatment groups, each having six pens and 10 piglets per pen. The feeding experiments were conducted over a period of 28 days. The groups were as below: control group (CON), fed with the basal diet; *B. licheniformis* group (BL), fed with the basal diet supplemented with 500 mg/kg *B. licheniformis* (HJ0135). The concentration of *B. licheniformis* products was 1 × 10^10^ CFU/gram of *B. licheniformis*. On day 29, twelve healthy piglets with similar body weights were selected from each group (a total of 24 piglets) and separated into two treatments per group. Six piglets in each group were intraperitoneally injected with LPS at a concentration of 100 μg/kg body weight, and the others were injected with sterile saline solution of the same volume. All the piglets were sacrificed 4 hours after the challenge with LPS. The LPS treatments were respectively labelled as LPS and BL-LPS, and the normal saline treatments were respectively labelled as CON and BL.

The experimental protocol was endorsed from the Ethics Committee of Zhejiang A & F University (Hangzhou, China). Basal diets were designed to meet the nutritional guidelines of the National Research Council (NRC; 2012) and were antibiotic-free (**Table 1**). Piglets were reared at Zhengxing Animal Husbandry Co., Ltd. (Hangzhou, China). Both water and feed were supplied *ad libitum*. Piglets were vaccinated following the farm’s routine vaccination program.

**Table 1:** Composition and nutrient levels of the basal diet.

Ingredients	Content, %	Nutrient level	Content
Corn	55.00	DE, MJ/Kg	14.17
Wheat midding	3.50	CP, %	20.35
Phospholipid	2.00	Lys, %	1.34
Whey powder	5.00	Met+Cys, %	0.77
Extruded soybean	7.30	Thr, %	0.80
Soybean meal	18.50	Ca, %	0.95
Fish meal	5.00	TP, %	0.65
Dicalcium phosphate	1.00	AP, %	0.48
Limestone	1.10		
NaCl	0.10		
L-Lysine HCl	0.35		
DL- methionine	0.15		
Vitamin-mineral premix^1^	1.00		
Total	100		

^1^Supplied the following per kg of diet: vitamin A, 10,000 IU; vitamin D3, 400 IU; vitamin E, 10 mg; pantothenic acid, 15 mg; vitamin B6, 2 mg; biotin, 0.3 mg; folic acid, 3 mg; vitamin B12, 0.009 mg; ascorbic acid, 40 mg; Fe, 150 mg; Cu, 130 mg; Mn, 60 mg; Zn, 120 mg; I, 0.3 mg; and Se, 0.25 mg.

### 2.2 Growth performance and diarrhea rate

Piglets were weighed on day 1 and 28 of this experiment to record their initial body weight (BW) and final BW, respectively. feed to gain ratio (F/G), average daily feed intake (ADFI) and average daily gain (ADG) were determined by recording the intake of piglets in each pen for the duration of the experiment. The total number of piglets with diarrhea was recorded each day during the entire experiment. The diarrhea rate was calculated as: total number of pigs with diarrhea/(total number of pigs × number of experiment days) × 100.

### 2.3 Sample collection

All 24 piglets were sacrificed following 4 h of LPS challenge to obtain blood, jejunal segment, jejunum mucosa, and colonic contents. The blood sample was collected from a jugular vein puncture, and the serum was gathered by centrifugation (12,000 × *g* for 10 minutes) at 4°C and kept at -20°C. Jejunal segments (approximately 1 cm) were fixed in 4% formaldehyde for the morphological assessment. Also, jejunum mucosa and colonic contents were immediately squeezed into a sterile tube and kept at -80°C for later analysis.

### 2.4 Serum antioxidant indexes

Serum antioxidant indices, i.e., superoxide dismutase (SOD), total antioxidant capacity (T-AOC), malondialdehyde (MDA) and glutathione peroxidase (GSH-Px), were assayed using kits commercially available from the Angle Gene Bioengineering Co., Ltd (Nanjing, Jiangsu, China).

### 2.5 Immune cytokine analysis

Levels of the immune-related indicators of serum and jejunum mucosa, namely, immunoglobulin (Ig)G, IgA, IgM, interleukin (IL)-6, IL-10, IL-1β and tumor necrosis factor (TNF)-α, were tested by the ELISA kits that obtained from the Angle Gene Bioengineering Co., Ltd (Nanjing, Jiangsu, China).

### 2.6 Jejunal morphology analysis

The fixed jejunal was dehydrated, embedded in the paraffin, sliced into sections (5 μm), stained using the hematoxylin-eosin, and finally sealed. The images were observed and acquired under a microscope. Six crypts and villi were chosen randomly in each section for detecting crypt depth and villus height under a 10 × magnification and their ratios were calculated.

### 2.7 VFAs analysis

Colonic VFAs (acetic, propionic, isobutyric, butyric, isovaleric, and valeric acids) were estimated using a gas chromatographic (GC) approach. Simply, 0.5 g of colonic contents were weighed and next mixed with pre-cooled pure water at a ratio of 1:2. The acquired mixture was mixed thoroughly for 30 seconds, left at 4°C for 30 minutes and subsequently centrifuged at 12,000g at 4°C for 10 minutes. The supernatant was mixed with phosphoric acid of 25% (m/v, 1:5) and left at 0°C for 35 minutes, followed by centrifugation at 10,000 g for 10 minutes at 4°C. The supernatant (500 μL) was filtered into a dedicated bottle for GC analysis.

### 2.8 Gene expression analysis by RT-qPCR

Total RNA was separated from the jejunal mucosa using TRIzol (Takara, Dalian, China) and was reverse transcribed into cDNA using RT reagent kit (Takara, Dalian, China). Real-time PCR was carried out on a CFX96 Touch instrument (Bio-Rad) with the SYBR Green PCR Master Mix (Takara, Dalian, China). The qPCR primers for the mRNA encoding the inflammasome proteins (*NLRP3*, *Caspase-1*, *IL-18*, *ASC*, *IL-1β*) are presented in **Table 2**. The levels of relative mRNA were assessed with 2^-ΔΔCT^ method.

**Table 2:** Real-time PCR primers.

Gene	Forward primer sequences (5’-3’)	Reverse primer sequences (5’-3’)
*β-actin*	CTACACCGCTACCAGTTCGC	TAGGAGTCCTTCTGGCCCAT
*NLRP3*	TTGTGTGGACCTATGCCCTG	GAGAGATGCAGCCCTTCTGA
*Caspase-1*	ATCAATTCGCACACGCCTTG	TATAGCCATGTCCGAAGCGG
*IL-18*	GCTGCTGAACCGGAAGACAA	AAACACGGCTTGATGTCCCT
*ASC*	GCTGGAATCAAAGCCCTTCC	TCCACGTCTGTGACCCTTGA
*IL-1β*	AAGATAACACGCCCACCCTG	GAGTTTCCCAGGAAGACGGG

### 2.9 Analysis of serum metabolomics

An acetonitrile/methanol (1:1, v/v) solution was incorporated into serum samples to obtain the metabolites. The mixture was vortexed for 30 seconds, sonicated at 4°C for 10 minutes and then left at -80°C overnight. On the second day, the samples were centrifuged at 12,000 rpm at 4°C for 15 minutes. The supernatant was debrided, and the pellet was desiccated after vacuum centrifugation. The samples were redissolved in acetonitrile/methanol mixture (150 μL, 1:1, v/v), sonicated at 4°C for 10 minutes, centrifuged at 12,000 rpm for 15 minutes and incubated at -80°C overnight. On the third day the supernatants were dissolved at 4°C and filtered through a nylon membrane (0.22 µm) into sample bottles for LC-MS/MS analysis.

Mass spectrometry conditions: capillary voltage: 3.5 kV positive ion, 3.5 kV negative ion, ion source temp: 325°C, the flow rate of drying gas: 10 L/min, atomization pressure: 35 psi, sheath temp: 370°C, gas flow rate in sheath: 12 L/min.

Raw data were processed using Agilent Profinder software to carry out retention time correction and identification, extraction, integration and alignment of peaks, etc., finally output CEF file. Statistical analysis was conducted using the Agilent Mass Profiler Professional (MPP) software, the Metlin database was also utilized for the identification of substances together with metabolic pathway analysis.

### 2.10 Statistical analysis

Comparisons between groups were made through one-way ANOVA utilizing SPSS software (version 25.0; SPSS Inc., USA), and statistical significance was set to *P* < 0.05. GraphPad Prism 8 software (GraphPad Prism Inc., USA) was exploited for developing histograms.

## 3 Results

### 3.1 Growth performance and diarrhea rate

**Table 3** reveals that *B. licheniformis* remarkably improved the ADG and final BW in contrast to CON group (*P* < 0.05). In addition, compared to control piglets, *B. licheniformis* dramatically reduced the F/G and diarrhea rate in piglets (*P* < 0.05).

**Table 3:** Effects of *B. licheniformis* on growth performance and diarrhea rate in piglets.

Item	Diet		SEM	*P*-value
	CON	BL		
Initial BW, kg	8.65	8.82	0.76	0.72
Final BW, kg	16.58^b^	18.45^a^	1.32	0.003
ADFI, kg	0.51	0.54	0.05	0.25
ADG, kg	0.28^b^	0.34^a^	0.037	0.001
F/G	1.78^a^	1.57^b^	0.18	0.01
Diarrhea rate, %	8.33^a^	5.85^b^	0.04	0.01

CON, control. BL, Bacillus licheniformis. BW, body weight. ADG, average daily gain. ADFI, average daily feed intake; F/G, feed/gain ratio. ^ab^Means within a row with different letters differ (P < 0.05). Values are expressed as the mean ± SEM, n = 6.

### 3.2 Serum antioxidant indexes

**Figure 1:**
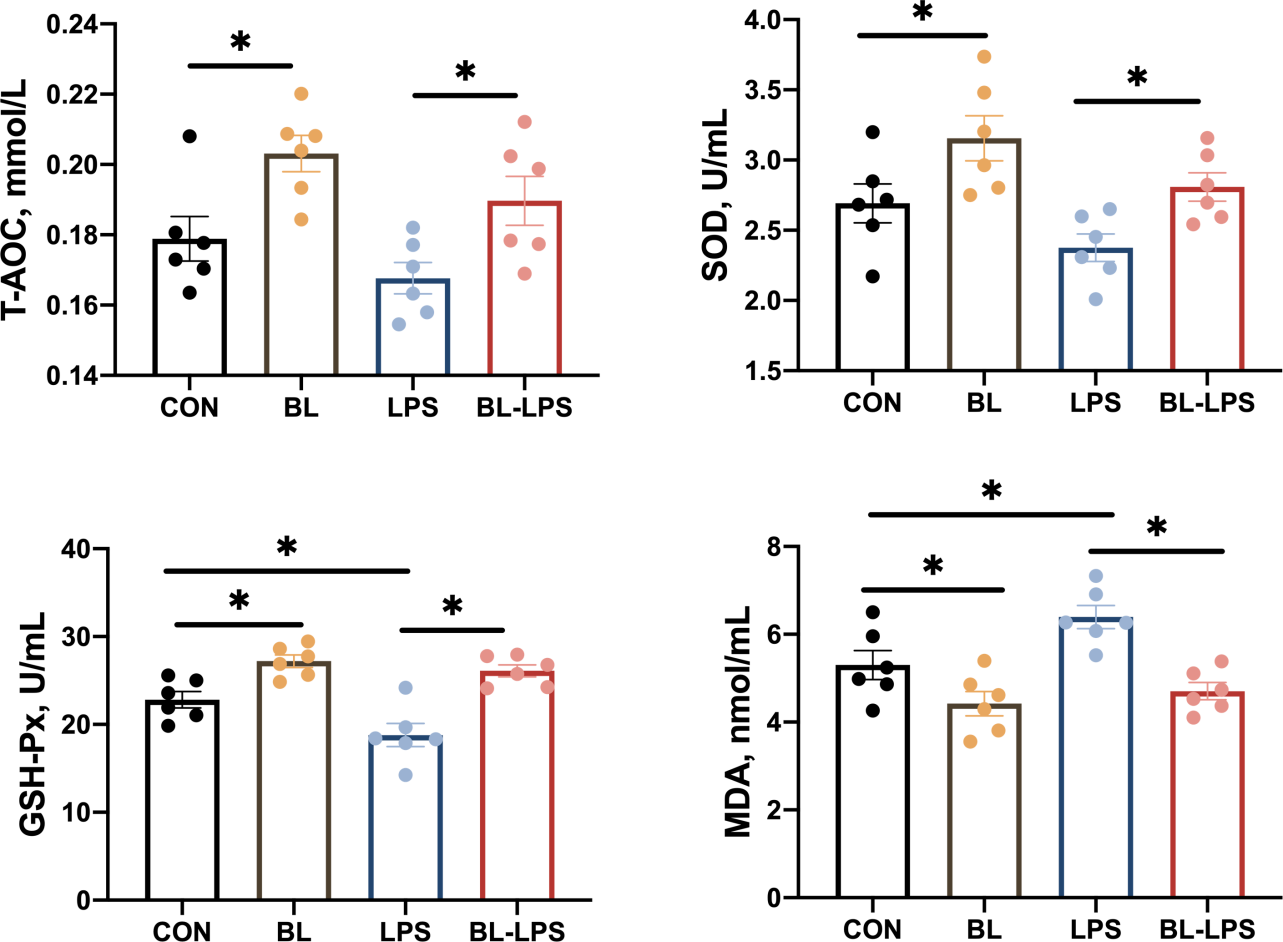
Effects of the *B. licheniformis* on serum antioxidant indexes of the weaned piglets challenged with LPS. CON: fed with the basal diet and injected with saline; LPS, fed with the basal diet and injected with LPS; BL, fed with the basal diet supplemented with *Bacillus licheniformis* and injected with saline, BL-LPS, fed with the basal diet supplemented with *Bacillus licheniformis* and injected with LPS. *Means significant difference (*P* < 0.05). Values are expressed as the mean ± SEM, n = 6.

In comparison to the CON group, GSH-Px, SOD and T-AOC activities increased significantly (*P* < 0.05), while the level of MDA decreased markedly in the BL group (*P* < 0.05). Piglets injected with LPS had lower GSH-Px activity and higher MDA content compared to the CON group. Adding *B. licheniformis* considerably reduced the serum MDA content and increased the GSH-Px, SOD and T-AOC activities compared to the LPS group (*P* < 0.05) (**Figure 1**).

### 3.3 Serum cytokines and immunoglobulins

**Figure 2:**
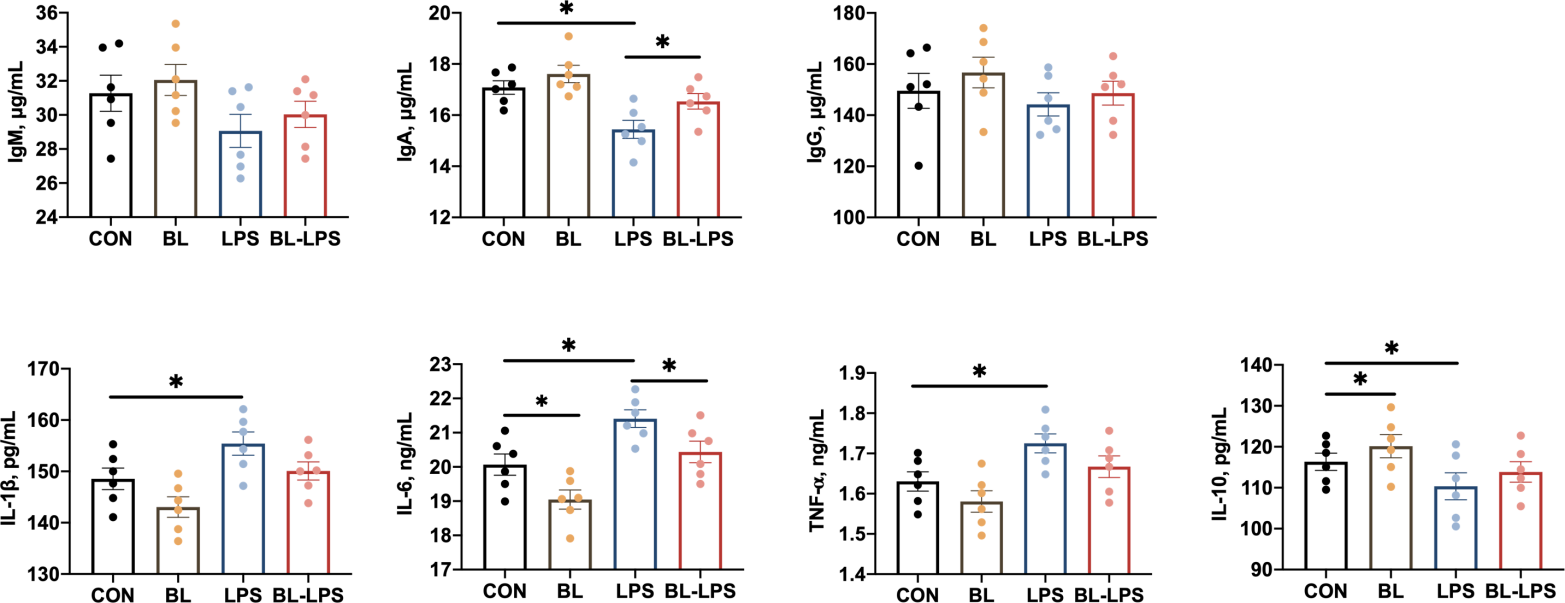
Effects of *B. licheniformis* on serum immune cytokines of the weaned piglets challenged with LPS. CON: fed with the basal diet and injected with saline; LPS, fed with the basal diet and injected with LPS; BL, fed with the basal diet supplemented with *Bacillus licheniformis* and injected with saline, BL-LPS, fed with the basal diet supplemented with *Bacillus licheniformis* and injected with LPS. *Means significant difference (*P* < 0.05). Values are expressed as the mean ± SEM, n = 6.

The impacts of *B. licheniformis* on the serum cytokines and immunoglobulins are presented in **Figure 2**. Compared to CON piglets, piglets injected with LPS showed lower IL-10 and IgA concentrations (*P* < 0.05) together with higher TNF-α, IL-6 and IL-1β contents (*P* < 0.05). While BL piglets showed lower IL-6 and higher IL-10 contents in their serum (*P* < 0.05). Additionally, in comparison to the LPS group, *B. licheniformis* considerably increased the level of IgA and reduced the IL-6 content (*P* < 0.05). However, the IgG and IgM values in the serum of all piglets did not differ significantly.

### 3.4 Morphological analysis of jejunum

**Figure 3:**
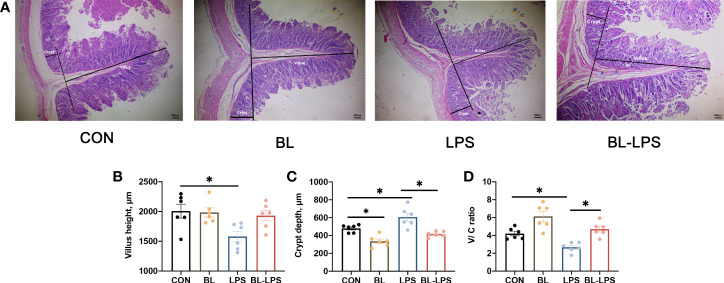
Effects of the *B. licheniformis* on jejunal morphology of the weaned piglets challenged with LPS. **(A)** The intestinal morphology of weaned piglets. **(B)** The villus height in the jejunum of weaned piglets. **(C)** The crypt depth in the jejunum of weaned piglets. **(D)** The ratio of the villus height and crypt depth. CON: fed with the basal diet and injected with saline; LPS, fed with the basal diet and injected with LPS; BL, fed with the basal diet supplemented with *Bacillus licheniformis* and injected with saline, BL-LPS, fed with the basal diet supplemented with *Bacillus licheniformis* and injected with LPS. *Means significant difference (*P* < 0.05). Values are expressed as the mean ± SEM, n = 6.

The results associated with jejunal morphology of the piglets are displayed in **Figure 3**. In contrast to CON group, *B. licheniformis* markedly attenuated the jejunum crypt depth (C), while injection of LPS significantly increased the crypt depth and reduced villus height (V) and V/C ratio (*P* < 0.05). The supplementation of *B. licheniformis* led to a reduction in the depth of jejunal crypts and an increase in the V/C ratio of piglets when compared to the LPS group (*P* < 0.05). *B. licheniformis* attenuated the decline in V/C ratio and inhibited the increase in crypt depth caused by LPS.

### 3.5 Jejunal mucosa cytokines and immunoglobulins

**Figure 4:**
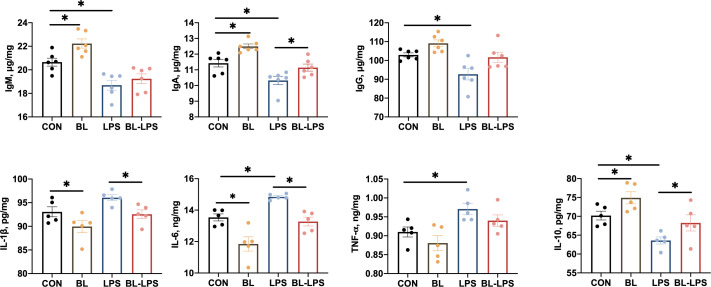
Effects of *B. licheniformis* on jejunal mucosa immune cytokine of the weaned piglets challenged with LPS. CON: fed with the basal diet and injected with saline; LPS, fed with the basal diet and injected with LPS; BL, fed with the basal diet supplemented with *Bacillus licheniformis* and injected with saline, BL-LPS, fed with the basal diet supplemented with *Bacillus licheniformis* and injected with LPS. *Means significant difference (*P* < 0.05). Values are expressed as the mean ± SEM, n = 6.

The impacts of *B. licheniformis* on the levels of cytokines and immunoglobulins on the jejunal mucosa are illustrated in **Figure 4**. In contrast to the CON group, the BL group showed significantly higher IgM, IgA, and IL-10 levels, and lower IL-6 and IL-1β contents (*P* < 0.05). The piglets injected with LPS showed noticeably lower levels of anti-inflammatory factors (IL-10) and immunoglobulins (IgG, IgA, IgM) (*P* < 0.05), and higher pro-inflammatory factors (TNF-α and IL-6) levels (*P* < 0.05). Furthermore, IL-10 and IgA contents were considerably higher (*P* < 0.05) and IL-6 and IL-1β levels were evidently lower (*P* < 0.05) after adding BL to the diet compared to the LPS group.

### 3.6 Expression of inflammasomes

**Figure 5:**
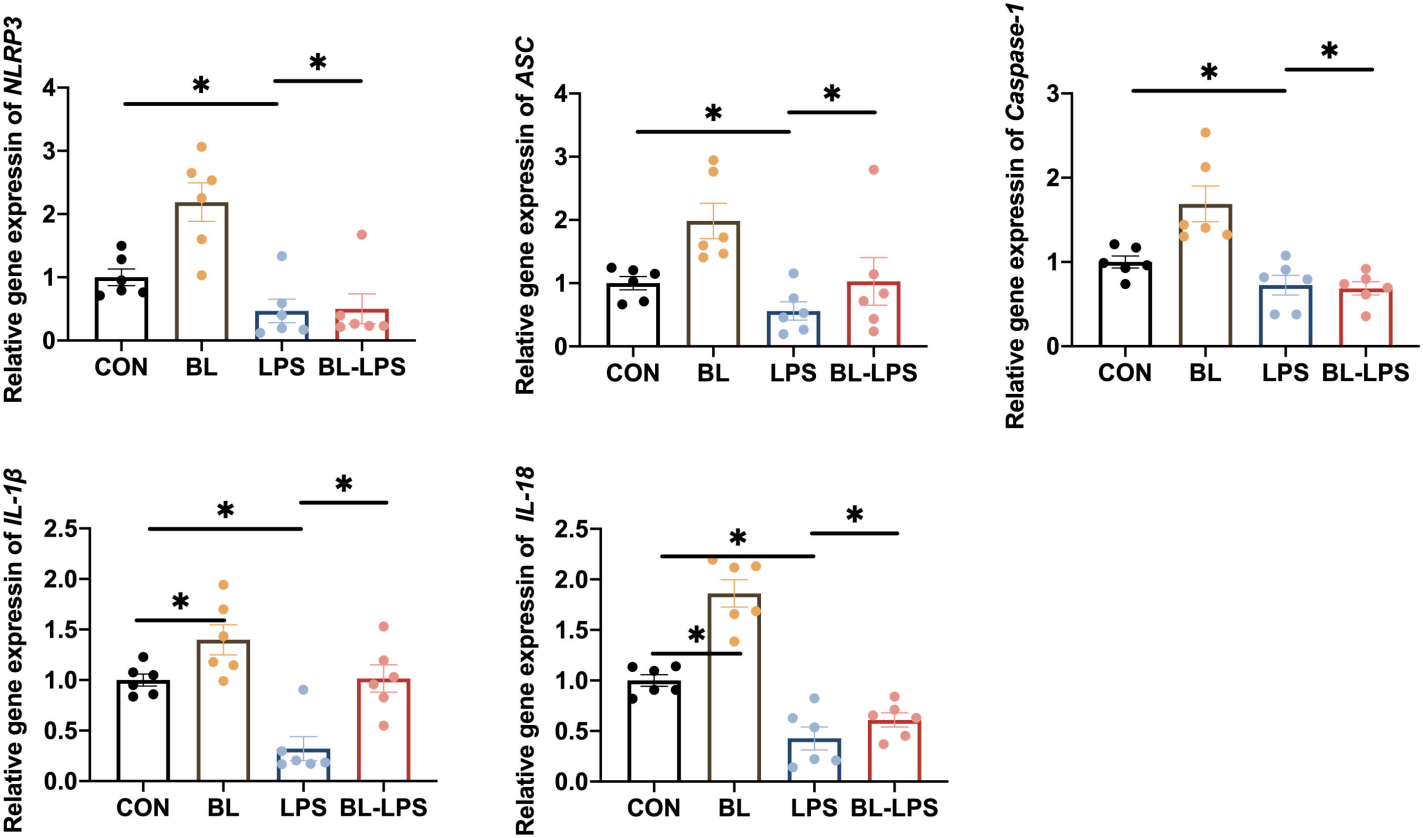
Effects of *B. licheniformis* on the inflammasomes in jejunum mucosa of the weaned piglets challenged with LPS. CON: fed with the basal diet and injected with saline; LPS, fed with the basal diet and injected with LPS; BL, fed with the basal diet supplemented with *Bacillus licheniformis* and injected with saline, BL-LPS, fed with the basal diet supplemented with *Bacillus licheniformis* and injected with LPS. *Means significant difference (*P* < 0.05). Values are expressed as the mean ± SEM, n = 6.

The impacts of *B. licheniformis* on the inflammasome in the jejunal mucosa in piglets are illustrated in **Figure 5**. It was revealed that LPS stimulation significantly increased the expression levels of *NLRP3*, *IL-1β*, *IL-18*, *ASC*, and *Caspase-1* in contrast to CON group, while *B. licheniformis* supplementation decreased the *IL-1β* and *IL-18* contents (*P* < 0.05). In comparison with LPS group, adding *B. licheniformis* to the diet could remarkably suppressed the levels of NLRP3 inflammasome complex genes (*P* < 0.05). In general, *B. licheniformis* alleviated LPS-induced activation of NLRP3 inflammasome.

### 3.7 Concentrations of VFAs

**Figure 6:**
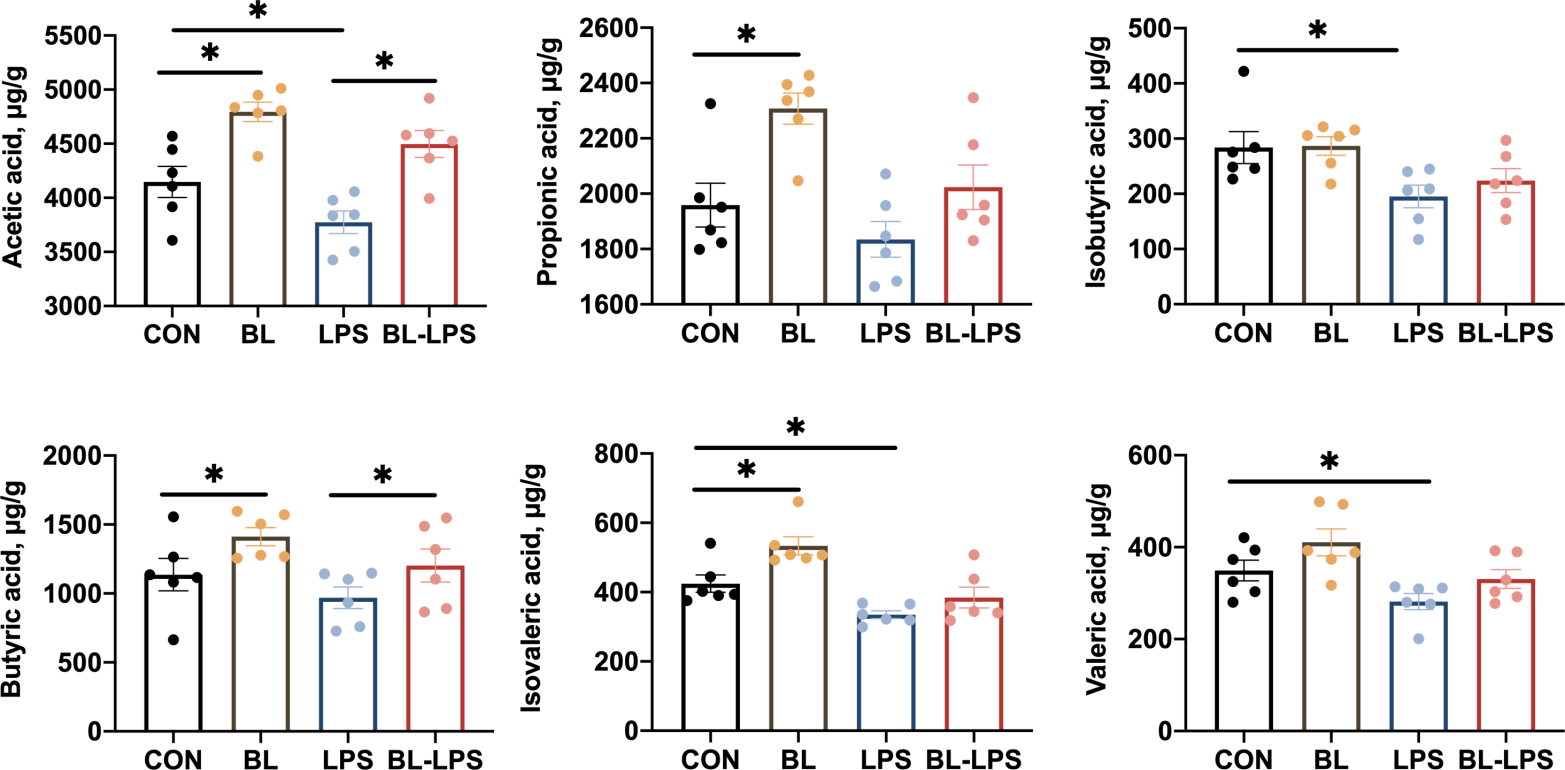
Effects of *B. licheniformis* on the colonic concentrations of VFAs of the weaned piglets challenged with LPS. CON: fed with the basal diet and injected with saline; LPS, fed with the basal diet and injected with LPS; BL, fed with the basal diet supplemented with *Bacillus licheniformis* and injected with saline, BL-LPS, fed with the basal diet supplemented with *Bacillus licheniformis* and injected with LPS. *Means significant difference (*P* < 0.05). Values are expressed as the mean ± SEM, n = 6.

The effects of *B. licheniformis* on the colonic concentrations of VFAs in piglets are depicted in **Figure 6**. The levels of propionic acid, acetic acid, isovaleric acid and butyric acid were markedly elevated in the BL group compared to those in the control group (*P* < 0.05). It was observed that the isobutyric acid, acetic acid, valeric acid and isovaleric acid levels were considerably lower in the LPS group compared to the control group (*P* < 0.05). Compared with the LPS group, the addition of BL upregulated the levels of acetic acid and butyric acid (*P* < 0.05).

### 3.8 Metabolome composition

**Figure 7:**
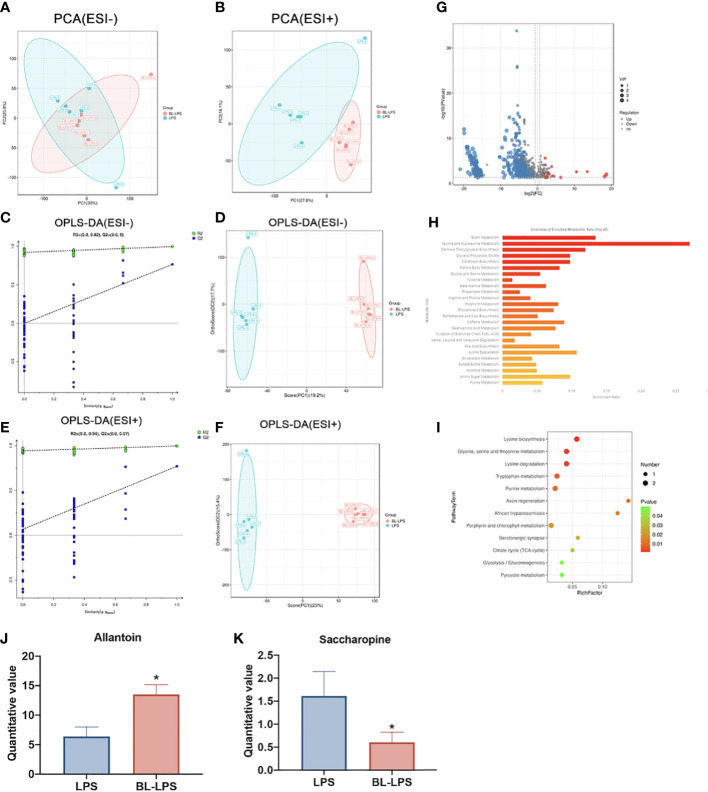
Effects of *B.licheniformis* on metabolome composition of serum in weaned piglets challenged with LPS. **(A, B)** Principal component analysis (PCA) score plot of nontargeted metabolite profiling of the serum samples among the two groups in both negative (ESI-) and positive ionization modes (ESI+). **(C)** The corresponding validation plot based on 200 times permutation tests demonstrated the robustness of the OPLS-DA model in a negative mode. **(D)** The score plot of OPLS-DA in a negative mode. **(E)** The corresponding validation plot based on 200 times permutation tests demonstrated the robustness of the OPLS-DA model in a positive mode. **(F)** The score plot of OPLS-DA in a positive mode. **(G)** The volcano map analyzed the differential metabolites in the serum. **(H)** KEGG pathway classification. **(I)** Bubble diagram showing the KEGG enrichment analysis. The bubble size indicates enriched the numbers, while the color shade indicates the differences. **(J, K)** Analysis of the differential metabolites by one-way ANOVA and Tukey’s test. LPS, fed with the basal diet and injected with LPS, BL-LPS, fed with the basal diet supplemented with *Bacillus licheniformis* and injected with LPS. *Means significant difference (*P* < 0.05). Values are expressed as the mean ± SEM, n = 6.

Multivariate analyses, including OPLS-DA and PCA, were conducted to identify clustering trends in the groups. The PCA score plot revealed that two groups had marked variations in serum metabolites both in ESI- (PC1 = 30%, PC2 = 20.8%) (**Figure 7A**) and ESI+ (PC1 = 27.80%, PC2 = 16.1%) (**Figure 7B**). Supervised mode identification of OPLS-DA demonstrated variations in category differentiation. **Figures 7D, F** presents the distinct changes in ESI- were noted in serum metabolism group (PC1 = 19.2%, OC2 = 17.7%) (**Figure 7D**), whereas ESI+ also showed complete separation (PC1 = 23%, OC2 = 15.4%) (**Figure 7F**). The findings of the 200 swap tests suggested that all the OPLS-DA models built were plausible, as both values of Q2 and R2 were lower as compared to original values (**Figures 7C, E**). The volcano map shows the expression of differential metabolites between the groups. A total of 162 differential metabolites were significantly upregulated and 736 were significantly downregulated (**Figure 7G**). Classification of the KEGG pathway indicated that the majority of serum compounds were engaged in metabolism, especially of taurine and hypotaurine metabolism (**Figure 7H**). The bubble diagram denotes the number of metabolites that are enriched in the signaling pathways of KEGG. The findings revealed 12 clearly enriched pathways, including lysine biosynthesis, glycine, serine, and threonine metabolism, lysine degradation, tryptophan metabolism, purine metabolism, axon regeneration, African trypanosomiasis, porphyrin and chlorophyll metabolism, serotonergic synapse, citrate cycle, glycolysis and pyruvate metabolism. (**Figure 7I**). Among the different metabolites, saccharopine was dramatically decreased, while allantion was evidently raised in the BL-LPS group compared to the LPS group (*P* < 0.05, **Figures 7J, K**). In addition, saccharopine was enriched in lysine biosynthesis and lysine degradation pathways. Allantoin was the most enriched metabolite in the purine metabolism pathway.

## 4 Discussion

In the last few years, several studies have demonstrated that probiotics can enhance the growth performance of animals, for example *B. subtilis* and *B. licheniformis* (22, 23). Daily oral supplementation with probiotics from birth to weaning can reduce the diarrhea rate in piglets (24). *B. licheniformis* can reduce the diarrhea score, improve performance, and decrease incidence and mortality of pre-weaning piglets (11, 25). The current results showed that adding *B. licheniformis* to diets markedly lowered the diarrhea rate and dramatically improved the growth performance of piglets in support of the previous research. *B. licheniformis* could enhance growth performance of chickens by increasing the activity of amylase in intestinal digesta (26). Zhang et al. indicated that *B. licheniformis* could enhance growth performance and minimize the diarrhea incidence in piglets (27). *B. licheniformis* has the potential to promote poultry growth (28).

Animal immune responses to infection are closely related to immunoglobulin levels. IgG, IgM, and IgA are the primary immunoglobulins that protect animals from infection. Cytokine secretion is usually the key to activating innate host defense systems and modulating the adaptive immune responses (29). The immune stress induced by LPS is primarily caused by the excessive production of pro-inflammatory cytokines, particularly TNF-α, IL-6 and IL-1β (30). IL-10 is an effective inhibitor of the secretion of IL-8, IL-1β and TNF-α induced by LPS. Consequently, IL-10 is regarded as an anti-inflammatory agent (31). Wang et al. (32) found that *B. licheniformis* reduced the pro-inflammatory cytokines expression (TNF-α and IL-1β) while raising the anti-inflammatory cytokines levels (IL-10 and IL-4). The combination of *B. licheniformis* and *Bifidobacterium* inhibited oxidative stress through attenuating the pro-inflammatory cytokines, for instance IL-18, IL-6 and IL-1β, while raising the anti-inflammatory cytokines, like IL-4 and IL-10 (33). The current study found that *B. licheniformis* supplementation decreased IL-6 level while increasing the IL-10 level in piglet serum. However, LPS stimulation decreased IL-10 and IgA levels and increased TNF-α, IL-6 and IL-1β contents in serum. In addition, our findings also indicated that pretreatment with *B. licheniformis* increased serum IgA, and decreased IL-6 level in weaned piglets challenged with LPS, suggesting that *B. licheniformis* had immune-promoting effects. Similarly, *B. licheniformis* pretreatment attenuated the serum levels of pro-inflammatory cytokines (namely, IL-1β and TNF-α) against acute liver injury induced by acetaminophen in rats (34). We speculated that *B. licheniformis* in the diet might regulate the immune responses of piglets by enhancing their serum immunity.

Serum antioxidant enzymes, for instance T-AOC, SOD, CAT, and GSH-Px, reflected the antioxidant capacity of the host, which worked together to eliminate excess free radicals and maintain homeostasis (35). Another important indicator of antioxidant capacity is MDA, the product of lipid peroxidation, which reflects oxidative stress (36). Jia et al. (37) observed that *B. licheniformis* could enhance the antioxidant capacity of fattening lambs, as evident by increased SOD and GSH-Px activities in serum. Zhao et al. (38) revealed that *B. licheniformis* H2 enhanced the antioxidative enzyme activities in the ileum and serum in broiler chickens challenged with *Clostridium perfringens*. We observed that *B. licheniformis* increased GSH-Px, SOD, and T-AOC activities, while decreasing the MDA content. The addition of *B. licheniformis* significantly reduced the MDA content and enhanced the GSH-Px, SOD and T-AOC activities in serum compared to the LPS group. Providing a diet containing *B. licheniformis* increased GSH-Px and SOD activities in the serum and mucus in tilapia (39). We hypothesized that *B. licheniformis* alleviated intestinal oxidative damage by clearing ROS. Among the differential metabolites measured using non-targeted metabolomics, we found that several metabolites were closely related to our antioxidant index results. Metabolomics showed that saccharopine content was reduced in piglets fed *B. licheniformis*. Saccharopine is a type of mitochondrial toxin and can cause mitochondrial dysfunction through its accumulation (40). Mitochondrial dysfunction can be relieved by enhancing antioxidant mechanisms (41). Additionally, Kong et al. (42) found that saccharopine was related to weight loss and inhibition of inflammation in mice. We found that allantoin content increased in *B. licheniformis* fed piglets. Allantoin is a naturally occurring and safe compound that has a marked impact on lipid metabolism, oxidative stress, and inflammation. Hamidi-zad et al. (43) found that allantoin enhanced the antioxidant activity by increasing SOD, CAT, and GSH levels. We speculated that *B. licheniformis* remarkably raised the antioxidant capacity of piglets by modulating the saccharopine and allantoin contents in the serum.

The intestine is a major component of nutrient absorption, and the health state of the villi is a pivotal factor in nutrient absorption (44). The integrity of the intestinal mucosa is fundamental for the proper function of epithelial cells and the prevention of intestinal toxins and pathogens from entering the bloodstream (45). Various stresses cause gastrointestinal barrier dysfunction, increase intestinal permeability to endotoxins, and lead to local and systemic inflammation (46). Lee at al (47). found that dietary supplementation with *B. subtilis* fermentation altered the morphology of the piglet’s small intestine, in particular the V/C ratio and the villi height. This suggested that probiotics improved intestinal health and digestive capacity of piglets. In our study, we also observed that LPS-induction raised crypt depth and reduced the V/C ratio, while *B. licheniformis* enhanced the intestinal health through lowering the crypt depth and raising the V/C ratio in weaned piglets. Deng et al. (48) observed that *B. subtilis* evidently enhanced the villus height and tended to raise the V/C ratio in ileum. The *bacillus* mixture decreased jejunum crypt depth and elevated the villus height and V/C ratio in the ileum and jejunum (49). These changes may be related to the fact that *B. licheniformis* increased the amount of VFAs in the colon, which stimulated the proliferation of intestinal epithelial cells and increased the villus height (50). Also, the increased villus height could promote the absorption of nutrients to improve the growth performance of piglets (51).

Impaired intestinal epithelial integrity may promote endotoxin invasion by intestinal microorganisms, leading to local imbalance of pro-inflammatory and anti-inflammatory molecules in intestine (52). Our current findings revealed that in contrast to CON group, *B. licheniformis* enhanced IgA and IgM levels, while LPS-induction decreased IL-10, IgA, IgM, and IgG levels, and elevated IL-6 and TNF-α levels in the piglets’ jejunal mucosa. Our findings displayed that *B. licheniformis* markedly inhibited the LPS-induced decline in IgA and IgG levels in the jejunal mucosa. In Cao’s study, the gene expressions of pro-inflammatory cytokines, like *IL-6*, *TNF-α*, *IL-1β* and *IL-8*, were shown to have increased in the jejunal mucosa of the piglets challenged with LPS (53). The NLRP3 inflammasome is a multi-protein compound that serves an essential function in innate immune system (54). NLRP3 is activated by LPS stimulation, NLRP3 recruits apoptosis-associated speck-like protein containing a caspase recruitment domain (ASC) and caspase-1 to form NLRP3 inflammasomes (55). In the inflammasome compound, caspase-1 is activated and initiated the pro-inflammatory cytokines release (IL-18 and IL-1β) (56). Nevertheless, no tests have been implemented to examine the impacts of *B. licheniformis* on the jejunal mucosal inflammasome in weaned piglets. We found that *B. licheniformis* restricted the activation of the NLRP3 inflammasome induced by LPS. Oxidative stress and proinflammatory processes have been considered to be interdependent, we hypothesized that *B. licheniformis* inhibited inflammation due to its increased antioxidant capacity. Metabolomics showed that allantoin content increased in piglets fed with *B. licheniformis*. Allantoin has been shown to alleviate lung inflammation and *TNF-α* gene expression levels (57), which is consistent with reduced serum and mucosal inflammation status of these piglets in the current study. Prior studies have indicated that saccharopine dehydrogenase is associated with the redox state and active inflammation (58, 59).

Microbes in the gut can also produce VFAs by breaking down the indigestible carbohydrates in food. These VFAs can suppress the development of certain harmful microorganisms, for instance *Salmonella* and *Escherichia coli* (*E. coli)* (60). VFAs, such as acetate, propionate, and butyrate, are important metabolites to maintain intestinal homeostasis and enhance intestinal barrier function (61). Treatments supplemented with *Lactobacillus plantarum* at suitable concentrations resulted in higher acetic acid and total VFA levels (62). *Lactobacillus* can produce lactic acid in the chicken lower intestine and convert it into VFAs, which may inhibit the growth of pathogenic bacteria in the intestine (63). In this study, *B. licheniformis* increased the concentration of VFAs after LPS challenge, suggesting that it could inhibit harmful bacteria and reduce inflammation. Many studies have shown that acetic, propionic, and butyric acids have immunomodulatory and anti-inflammatory impacts *in vivo* and *in vitro*. For instance, VFAs play important roles in homeostasis because of their metabolic and immunomodulatory effects (64). Butyrate, propionate, and acetate suppress TNF-α-induced inflammatory responses in Caco-2 cells and the mouse colon (65). Butyrate and propionate can suppress the LPS-induced cytokines expression like IL-12 and IL-6, displaying potent anti-inflammatory properties (66). The fermented product of *Bacillus licheniformis* could increase propionic acid content in the cecal digesta of broilers challenged by coccidia (67). Wang et al. (68) observed that *B. licheniformis* significantly increased the content of acetic acid and butyric acid contents in the feces of growing pigs. VFAs regulate macrophage production of inflammatory mediators. Cox et al. (69) reported the attenuation of IL-10 production by monocytes treated with VFAs. These results demonstrated that *B. licheniformis* might produce VFAs to relieve inflammation caused by LPS stimulation.

## 5 Conclusion

**Figure 8:**
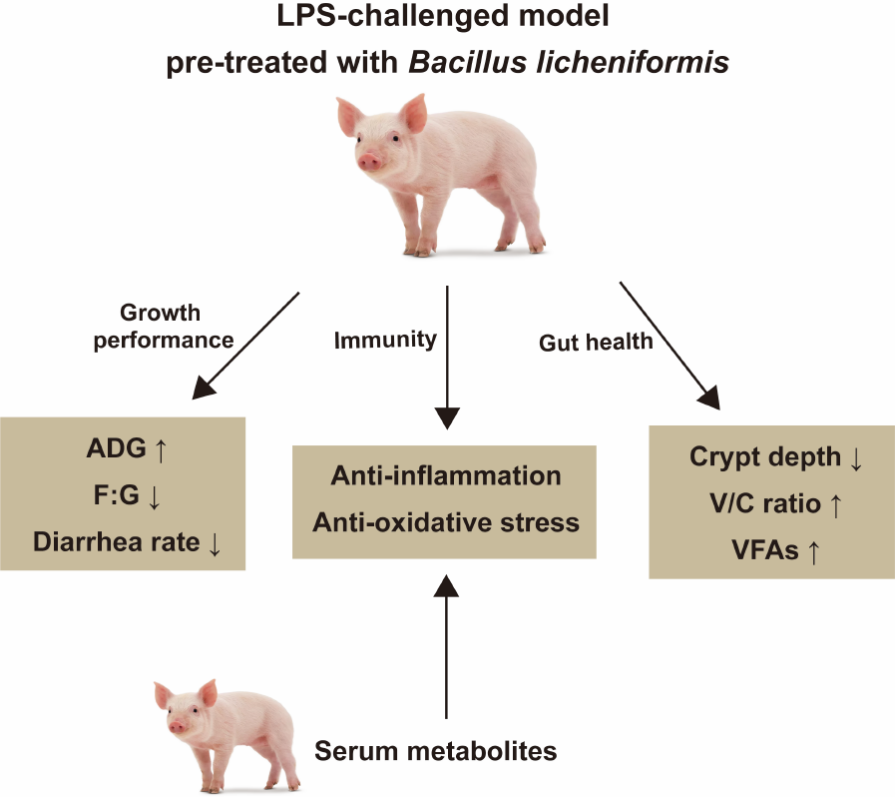
The summary of the study. *B. licheniformis* improved growth performance, immune function, and intestinal health of weaned piglets.

In summary, this study showed that *B. licheniformis* improves growth performance in weaned pigs and *B. licheniformis* provides protection against LPS-induced inflammation and injuries in the weaned pigs, probably by strengthening the immune response and intestinal barrier, regulating the NLRP3 inflammasome, altering VFA production, and modulating serum metabolism. **Figure 8** summarizes the results presented. The above results indicate the potential usefulness of *B. licheniformis* in safeguarding intestinal health.

## Data availability statement

The original contributions presented in the study are included in the article/supplementary materials. Further inquiries can be directed to the corresponding author/s.

## Ethics statement

The animal study was reviewed and approved by the Ethics Committee of Zhejiang A & F University.

## Author contributions

CY designed and supervised the study. XY drafted the manuscript. ZD, ZC, and RZ conducted the experiments. XY and ZD performed data analysis. CY and YX revised the manuscript. All authors contributed to the article and approved the submitted version.
